# Fast Antioxidant Reaction of Polyphenols and Their Metabolites

**DOI:** 10.3390/antiox10081297

**Published:** 2021-08-17

**Authors:** Janusz M. Gebicki, Thomas Nauser

**Affiliations:** 1 Department of Biological Sciences, Macquarie University, Sydney 2109, Australia; 2 Departement für Chemie und Angewandte Biowissenschaften, Eidgenössische Technische Hohschule, 8093 Zürich, Switzerland; nauser@inorg.chem.ethz.ch

**Keywords:** oxidative stress, antioxidants, free radicals, polyphenols, kinetics, reaction mechanism, radical adducts

## Abstract

The negative correlation between diets rich in fruits and vegetables and the occurrence of cardiovascular disease, stroke, cancer, atherosclerosis, cognitive impairment and other deleterious conditions is well established, with flavonoids and other polyphenols held to be partly responsible for the beneficial effects. Initially, these effects were explained by their antioxidant ability, but the low concentrations of polyphenols in tissues and relatively slow reaction with free radicals suggested that, instead, they act by regulating cell signalling pathways. Here we summarise results demonstrating that the abandonment of an antioxidant role for food polyphenols is based on incomplete knowledge of the mechanism of the polyphenol-free radical reaction. New kinetic measurements show that the reaction is up to 1000 times faster than previously reported and lowers the damaging potential of the radicals. The results also show that the antioxidant action does not require phenolic groups, but only a carbon-centred free radical and an aromatic molecule. Thus, not only food polyphenols but also many of their metabolites are effective antioxidants, significantly increasing the antioxidant protection of cells and tissues. By restoring an important antioxidant role for food polyphenols, the new findings provide experimental support for the advocacy of diets rich in plant-derived food.

## 1. Oxidative Stress and Human Health

There is little doubt that the desire to identify the causes of human disease is a major motivation driving much biochemical research. Successful solution of the many problems revealed is most commonly achieved by a combination of research talents in borderless collaboration of skilled individuals with common interests, but with varied experience. An essential component of the most effective collaborations is the pursuit of both basic and applied research, a principle well illustrated by the outstanding scientific career of Dr Umberto Dianzani, who, in addition to personal experience as a haematologist, pathologist, pharmacist, chemist and biochemist, was a key member of extensive collaborative research with many groups in many countries [1]. One of Professor Dianzani’s major interests was the biological damage by free radicals and its prevention by antioxidants. In this review, we continue this interest by summarising recent results demonstrating the potential of plant-derived food components and their metabolites to protect humans exposed to oxidative stress.

In common with other aerobic organisms, humans are constantly subjected to damage from reactions involving oxygen or its partially reduced derivatives, commonly designated as reactive oxygen species, or ROS. To cope with the challenge, we are equipped with an arsenal of antioxidant defences made up of specialized enzymes and small molecules: the former are made up of superoxide dismutase, catalase, peroxiredoxin and other peroxidases, while the principal endogenous small antioxidants are vitamins A, C and E, glutathione and other thiols, urate and metal ion chelators. Under normal conditions, this combined system can cope with the oxidative challenge. However, there are conditions, particularly associated with modern industrial life, which can greatly increase the oxidative challenge: they include radiation, pollutants, drugs and other xenobiotics, injury and many other agents [2,3]. When the oxidative challenge is high, the organism can be in a state known as Oxidative Stress (OS), originally defined as a condition in which an organism’s antioxidant defences are insufficient to prevent or repair molecular damage caused by ROS [4]. This simple definition was later extended to include disruption of redox signalling [5], but in this review we use the original version because it emphasizes the phenomenon of biomolecular damage, potentially resulting in health impairment. The range of diseases and other deleterious conditions apparently associated with OS is extensive: it includes various forms of cancer, cardiovascular disease, conditions associated with ageing, inflammation, disorders of the immune system, obesity and diseases of the lung, kidney, liver, eye, brain, muscles, bones and other tissues [4,6,7,8,9,10]. While future studies may show that the currently accepted list of the deleterious health conditions includes some not associated with oxidation, there is little doubt that OS is a health hazard.

Oxidative stress is commonly coupled with excessive formation of free radicals, with some researchers actually identifying free radicals as the origin and cause of OS. Free radicals are atoms, molecules or ions with one or more unpaired electrons. The most reactive ones have high reduction potentials; i.e., they readily oxidize most molecules indiscriminately. There is a common misconception that all free radicals are highly reactive, but in fact the range of those able to cause biological damage is quite narrow: it is made up principally of hydroxyl, peroxyl, alkoxyl, thiyl, phenoxyl and semiquinone free radicals, and high valence transition ions. The usual form of damage is abstraction of an electron or H atom from a target molecule, creating a new, less reactive secondary target free radical. In complex biological systems, the result is a chain of successive electron transfers, creating new free radicals with decreasing reactivity, as required by thermodynamics. If the chain involves critical molecules, such as DNA, proteins or lipids repaired or replaced only slowly, the result may be a permanent impairment of a vital function, which, if not reversed, can constitute the first step in the development of a disease or another form of damage. The radical chain is only terminated by reaction with another free radical, a transition metal ion or with an antioxidant, with the last creating a radical no longer able to propagate the damage.

In discussing the phenomenon of oxidative reactions of free radicals and their consequences, it has to be noted that low levels of free radicals form continuously in vivo and fulfil significant roles in tissue defence, DNA biosynthesis, redox regulation and possibly in thiol-based cell signalling [2,11,12,13,14,15]. There is therefore a clear and important distinction between the consequences of formation of low and excessive levels of free radicals; the former are essential and not a danger to the organism because its antioxidant defences can prevent or repair any collateral molecular damage, while the latter can trigger or aggravate a wide range of diseases and other undesirable conditions.

## 2. The Principal Reactions of Free Radicals In Vivo

**Scheme 1:**
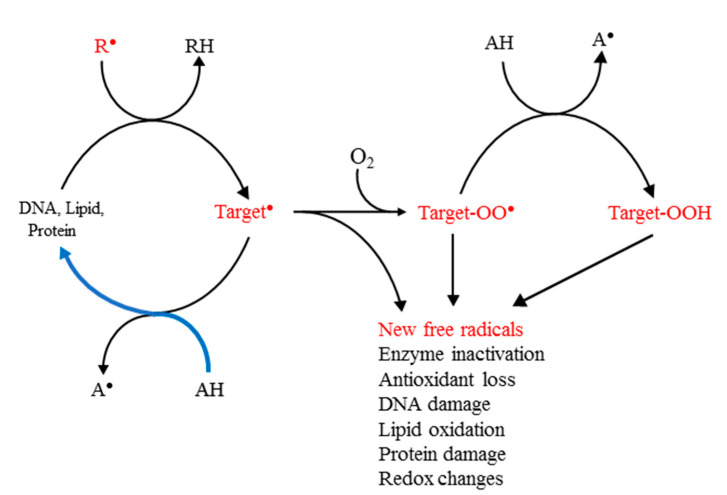
Principal biological targets of the primary free radicals and damage repair. R^●^ is the initiating free radical and AH an antioxidant. The initial reaction produces a carbon-centred free radical in the target. Reactive species are shown in red. The blue curve shows the flow of the reducing equivalents under optimal prevention of damage.

The initial reaction of the primary damaging free radicals with biomolecules commonly produces carbon-centred (C-centred) free radicals. Their principal subsequent reactions result in new free radical chains, which include formation of peroxyl free radicals, ROO^●^, in a fast reaction with physiological oxygen (Scheme 1).

In the reducing environment of cells and tissues, peroxyl radicals are commonly converted to hydroperoxides. All these species, namely, the C-centred and other free radicals, peroxyl radicals and hydroperoxides, have the capacity to propagate the damage triggered by R^●^, with peroxyl radicals believed to be the main carriers of damage in living organisms; many of the commonly used assays for the formation of free radicals in cells and tissues depend on the detection of peroxyl radicals and hydroperoxides [16,17].

Defence against free radical-induced damage can be achieved by scavenging the R^●^ or repair of the damaged target molecules. Under oxidative stress, this should be achievable in theory by enhancing the levels of the endogenous antioxidants or, if necessary, supplementation with additional antioxidants. Considerations of the mechanism of action of the most damaging free radicals, such as the hydroxyl, HO^●^, have shown that, because of their high reactivity and low levels in vivo, direct scavenging by added radical scavengers is not feasible, despite a commonly held contrary view [18]. This means that the earliest and most effective point for protective action is the secondary target free radical, because its repair prevents subsequent molecular damage (Scheme 1). It is important to note that the well-documented recognition of the signalling and defensive functions of ROS in vivo means that the role of antioxidants in living organisms is not to eliminate all free radicals, but rather to lower any excessive levels, so that they can be neutralised by the endogenous antioxidants present.

The practical possibility of modification of biological damage by antioxidants is based on extensive chemical knowledge of the properties of free radicals, which established that their effectiveness is largely determined by thermodynamics and kinetics [19,20]. The former is an intrinsic property of the reacting species and cannot be altered. Importantly, however, kinetic factors can be manipulated because they depend on concentrations of the reactants:

Rate of reaction = k [AH] [R^●^] (1)

In the equation, k is the bimolecular rate constant (units M^−1^ s^−1^), AH an antioxidant and R^●^ a free radical, with the bracketed terms denoting molar concentrations. Since the damage-causing primary R^●^ cannot be scavenged in vivo and are by definition sufficiently reactive to generate C-centred free radicals in many molecules, antioxidant repair reactions must be faster than those of any competing processes. This cannot be achieved under OS by the normal levels of the endogenous antioxidants. In fact, measurements of the rate constants of reactions of ascorbate, urate and GSH with C-centred radicals have shown their reactions to be too slow to ensure effective scavenging of the radicals in tissues with a low antioxidant content [21,22,23]. The obvious defensive tactic of increasing the levels of lthe endogenous antioxidants to levels sufficient to prevent the consequences of OS is not possible: in the case of enzymes and metal-chelating proteins, their activities are tightly regulated and are not amenable to manipulation in human populations. Similarly, rates of free radical repair by the principal non-enzymatic antioxidants are not readily enhanced, because their concentrations in vivo cannot be significantly increased in humans consuming a normal healthy diet. This possibility was extensively studied for the vitamins C and E and may well be the reason for the generally disappointing benefits of treatment with vitamins and other supplements found in several long-term studies of thousands of subjects [4,24,25,26,27]. There is likely to be a limit to the effective in vivo concentrations of many added supplements: experimental evidence shows that the plasma concentration of the excellent antioxidant ascorbate reaches a limit at a modest oral intake of the vitamin. [28].

The inadequacy of the normal human antioxidant defences under OS, demonstrated by the population and theoretical results, shows that additional antioxidants are required by many individuals. These should persist in tissues and be easily administered, preferably by diet. Currently, the most promising candidates for this role are the plant-derived food flavonoids and other polyphenols, and many of their metabolites.

## 3. Food Polyphenols and Health

For several decades, dietary polyphenols have been the subject of intense research activity because of their perceived beneficial role in human health. Currently, the health-food polyphenol link results in over 6500 entries in the Medline database and the number of publications is increasing [29]. Polyphenols form a diverse group of over 8000 chemically characterised secondary metabolites in plants, with about 500 commonly consumed as part of the human diet, where they are particularly abundant in vegetables, fruit, red wine, berries, green tea, soy and cocoa [17,30,31]. Many epidemiological and interventionist studies have been conducted in attempts to disclose convincing links between polyphenol intake and human health. Epidemiological research examined the relationship between diets and conditions such as mortality, cancer, atherosclerosis, cardiovascular disease, diabetes, inflammation, ischemia, hypertension, stroke, metabolic syndrome, obesity, urinary tract infections and conditions associated with ageing, such as cognitive degeneration and Alzheimer’s and Parkinson’s diseases. Such studies are long, expensive and fraught with methodological difficulties, but have the capacity to provide convincing links between diet and disease. The broad overall findings were that a polyphenol-rich diet had either a preventive or alleviating effect on the condition, or produced no detectable change.

In intervention studies, groups of subjects ranging from a few to over 100 were administered a course of single or several food polyphenols for various periods and the effect on selected biomarkers compared with unsupplemented controls. The results of 93 intervention studies with both healthy and health-impaired subjects, conducted before 2005, showed some protective effects on the plasma antioxidant biomarkers, vascular system and on markers of carcinogenesis, with the authors pointing out the design limitations of many of the studies [32]. One common problem was the inclusion of healthy individuals; by definition, endogenous defences are normally capable of providing adequate antioxidant protection for healthy subjects, so that studies of the effects of diets rich in polyphenols are much more likely to produce measurable results in subjects suspected or known to be exposed to excessive free radical challenge; i.e., to OS. There is, in fact, evidence that the effectiveness of dietary interventions on plasma biomarkers is much greater in individuals compromised by disease than in healthy controls [33,34]. While studies of persons known or suspected of exposure to OS demonstrated a general tendency towards a reduction in the consequences of the stress and enhancement in antioxidant potential by polyphenol administration, many results are confusing and sometimes self-contradictory [35]. One aspect of the results is significant; however, as far as we know, there were no reports of increased oxidative damage in the groups consuming plant-derived food, as would be expected if the ingested polyphenols had a pro-oxidant effect.

The results and conclusions drawn from a wide range of studies have been summarized in many comprehensive reviews published in the last decade, constantly updated because of the rapid increase in the numbers of studies of the effects of plant-derived food on health. Those quoted are selected to give an overall view of the current state of the field, with the list constantly expanding and limited here only by space requirements [36,37,38,39,40,41,42].

## 4. New Developments in the Antioxidant Role of Polyphenols

Given the generally accepted health benefits of food polyphenols, the intensive search for their cause is not surprising. An early theory, based on the long-known ability of phenolic compounds to act as chemical antioxidants, acquired physiological relevance following the discoveries of the presence of polyphenols in human diet and of the generation of free radicals in living organisms. While the antioxidant theory was accepted for several decades, recent re-examination of the beneficial role of dietary polyphenols on health suggested that their physiological effects are more likely to be due to their interaction with macromolecules and the ability to affect the cell redox signalling functions. [12,13,34,35]. The virtually universal abandonment of a major antioxidant role for food polyphenols in vivo was based on three sets of observations: (1) the polyphenol reaction with free radicals was believed to be too slow for successful competition with oxygen and ascorbate; (2) modification of the redox properties of polyphenols by extensive metabolism, including the loss of phenolic groups, would not allow them to function as effective antioxidants; and (3) the concentrations of the ingested polyphenols in biofluids are too low for effective antioxidant action [12,17,31,43].

These conclusions are now challenged by reports indicating that the dismissal of an antioxidant role of polyphenols in vivo is incorrect, because it is based on incomplete knowledge of the mechanism of reaction of polyphenols with free radicals. The classical mechanism assumed that antioxidant action required transfer of electrons from the polyphenol to the radical. However, new experimental results show that (1) the polyphenol reaction with radicals is very fast and lowers their damaging potential; (2) loss of phenolic groups does not abolish the antioxidant ability of the polyphenols; and (3) the antioxidant defences of an organism include both polyphenols and their aromatic metabolites, present in vivo at µM concentrations. Evidence for these conclusions is summarised below.

### 4.1. Speed of the Polyphenol-Free Radical Reactions

The classical definition of oxidation is the “complete, net removal of one or more electrons from a molecular entity” [44]. Thus, in a single electron transfer involving a free radical R^●^ and a polyphenol electron donor PPOH, the donor is oxidized to a free radical PPO^●^. This, in an environment rich in reducing compounds, AH, such as a cell, regenerates the donor and produces a new free radical A^●^ with diminished reactivity. As radical reactions go, the overall process is relatively slow, because of a large entropic barrier, since it involves the exchange of at least two protons and breaking of the strong O–H bond [45]:

R^●^ + PPOH → RH + PPO^●^ (2)

PPO^●^ + AH → PPOH + A^●^ (3)

Only a few studies addressed the kinetics of reactions of flavonoids and other phenols with C-centred free radicals. Experimental investigations required the employment of pulse radiolysis, with accelerated electrons generating a C-radical, R^●^, which then reacted with the phenolic compound [46]. Measurements of the subsequent decay of the R^●^ or formation of PPO^●^ allowed the derivation of the speed of the reaction by calculation of the reaction rate constant (Equation (1)). The results of early studies with polyphenols are shown in the first seven k_f_ values listed in Table 1. Apart from one value, the rate constants were less than 10^8^ M^−1^ s^−1^. This is much lower than the 10^9^–10^10^ M^−1^ s^−1^ range common for free radical reactions, termed “diffusion controlled”, because they require little or no activation energy and their speed is primarily limited by diffusion of the reactants. The rate constants of Reaction (2) confirm that, according to the classical mechanism of oxidation, polyphenols cannot compete with the parallel fast reactions of the DNA, protein or lipid C-centred free radicals with physiological oxygen or ascorbate.

In a new development, the possibility of a very fast reduction in the damaging potential of free radicals by polyphenols was suggested by a report of rapid scavenging of alcohol C-centred radicals by histidine [49]. In this study, aqueous solutions containing an alcohol and a low concentration of histidine were irradiated with 50 ns pulses of 2 MeV electrons, with the formation of transient absorbing species followed optically. The kinetics of absorbance changes were measured on the µs time scale. In solutions saturated with N_2_O, the electron pulse decomposed the solvent water, producing virtually exclusively the powerful oxidizing hydroxyl free radicals HO^●^ [19]. The HO^●^ generated C-centred alcohol free radicals, which formed adducts with the His in a reversible reaction with a forward rate constant >10^9^ M^−1^ s^−1^, or about 1000 times faster than the classical antioxidant reaction involving electron and/or H transfer (Table 1). This result was supported by measurements of the forward rate constants of reactions of His or nitrobenzenes with C-centred radicals from isopropanol, CO_2_^−^, CF_3_ and CCl_3_, which were virtually diffusion-controlled, with values between ≤3 × 10^9^ and 2 × 10^10^ M^−1^ [50,51,52].

Analysis of these reports showed that the only requirement for a molecule forming an adduct with the free radical was the presence of an aromatic centre. Since flavonoids and other polyphenols, and many of their metabolites, have such centres, tests were carried out to determine their potential to form adducts with amino acid C-centred radicals generated by pulse radiolysis [48]. Acetylated amino acid derivatives of Lys, Pro, Gly, Glu and cyclo(Gly)_2_ were used, because of their structural relevance to peptides and proteins. The concentration ratio of the amino acid *N*-Ac-AA-NH_2_ to polyphenol was 200:1, ensuring that 98% of the HO^●^ reacted with the amino acid, leaving the polyphenol unaffected:

*N*-Ac-AA-NH_2_ + HO^●^ → *N*-Ac-AA^●^-NH_2_ + H_2_O (4)

In the absence of oxygen, the amino acid free radicals formed adducts with the polyphenols in a reversible process. For morin, the reaction was

*N*-Ac-AA^●^-NH_2_ + morin ⇌ [adduct]^●^ (5)

While morin was used in most experiments, similar fast reactions were recorded with the flavonoids rutin and epigallocatechin gallate and with the polyphenol metabolite gallate (Table 1). Further details of the formation and characteristics of the free radical–polyphenol adducts were elaborated in a subsequent publication [53].

It is clear that the large differences in the two groups of low and high rate constants, and therefore the speeds of the polyphenol-C-radical reactions, were determined by the mechanism of the process. When the electrons were transferred, as in Reaction (2), the process was slow, for the reason given above. When an adduct formed without electron transfer, the reaction was practically diffusion controlled. Since some of the earlier studies of polyphenol free radicals also employed pulse radiolysis, an obvious question is why they failed to observe formation of the radical adducts [46,54,55]. The reason is that only Filipe et al. have studied the reaction between C-centred free radicals and a polyphenol flavonoid, measuring the kinetics of reaction of Trp^●^ and catechin on a time scale too slow to observe the adduct.

From the point of view of biological protection, it is important to note that the first step in the reaction resulting in formation of the free radical–polyphenol adduct is already an antioxidant event, because it results in significant reduction of the reactivity of the C-centred radicals, as required by thermodynamics.

### 4.2. The Antioxidant Role of OH Groups in Polyphenols

The role of antioxidants is to reduce the damaging potential of ROS. While the classical free radical scavenging action of polyphenols required the transfer of a hydrogen from a phenolic group [45], the results summarised in Section 4.1 demonstrate that this group is not necessary for fast formation of an adduct; the only requirements are a C-radical and an aromatic compound. This finding greatly expands the range of available dietary antioxidants, because the requirements for adduct formation are fulfilled by a plethora of polyphenol metabolites, as discussed in Section 4.4.

### 4.3. The Principal Reactions of C-Radicals In Vivo

**Scheme 2:**
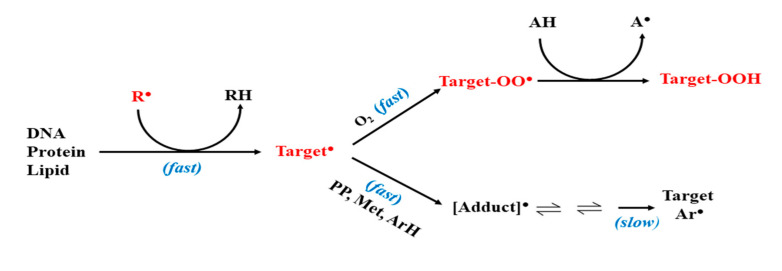
The major competing reactions of C-centred free radicals under physiological conditions. Intermediates capable of causing molecular damage are in red. PP is a polyphenol, Met an aromatic metabolite and ArH an aromatic molecule.

The experimental observations of the formation and subsequent fate of the adducts are consistent with the processes illustrated in Scheme 2. In this, [adduct]^●^ formation is assumed to be followed by a series of equilibria, with the final slow transfer of electrons in Reaction (2). The different values of the reaction rate constants reported in Table 1 can be explained by the time point at which the reaction was measured; adduct detection required measurements on the low μs scale, while in the earlier studies only the final slow stage involving electron or H transfer was seen (Reaction 2).

### 4.4. Concentration of Polyphenols and Their Metabolites In Vivo

Knowledge of the tissue concentration of any bioactive antioxidant is important because, among other reasons, of its influence on the rates of reactions (Equation (1)). Measurements of the concentrations of flavonoids and other polyphenols in humans typically involved oral administration of the selected compound, followed by its detection in plasma and urine at different times [56,57]. The results can give the maximum biofluid concentrations of the parent compound (C_max_) and times of its clearance (T_max_). Studies of the ability of the administered polyphenol to affect a particular health-related parameter commonly compare its effects in normal controls and in patients with an identified condition, such as cancer or neurological disease. With some exceptions, the general results of the treatments have proved beneficial. However, usually the benefits cannot be quantitatively related to levels of the polyphenol in plasma or urine, because accurate values of their concentrations are difficult to derive. For example, the commonly used LC-MS technique shows significant variability in interlaboratory results, sometimes differing by a factor of 10 [58,59].

In spite of such problems, already existing data allows the drawing of some broad conclusions. Analysis of 424 intervention studies with polyphenol-rich food sources and pure polyphenols led Rothwell and collaborators to conclude that a plasma concentration of over 5 µM should be achievable by simultaneous consumption of a variety of foods rich in several polyphenols, as is common in daily life [30]. Over 380 metabolites were identified in the Phenol-Explorer database, which, together with the parent compounds, form part of the total phenolic metabolome of human tissues. Many of the metabolites retained the aromatic character of their parent polyphenols and showed higher T_max_ values. The authors implied that, however valuable, measurements of the levels and persistence of singly administered polyphenols in biofluids are not useful for estimating the total contribution of a vegetable-rich diet to health, because the effects of the individual dietary components are additive. The derived consensus was that the physiological concentration of the total polyphenol-derived metabolome in humans varies widely and can reach µM concentrations, but is unlikely to exceed 10 µM [30,32].

The contribution of these levels of dietary polyphenols to the organism’s antioxidant potential needs to be augmented by the discovery that not only polyphenols but also other aromatic food components can lower the damaging potential of C-centred free radicals by forming adducts. Recent work has shown that a major source of aromatic food metabolites are the non-extractable polyphenols, including condensed tannins, ellagitannins, flavanone rutinosides and other high molecular weight polyphenols, not soluble under conditions usually employed for a polyphenol assay in tissues, but requiring previous acid hydrolysis [60,61,62]. The condensed polyphenols form a large proportion of the phenolic content of many plant-derived foods; for example, studies with apples, nectarines and pears showed 5 times more non-extractable than extractable polyphenols by weight [62]. The large molecules are not absorbed in the stomach or small intestine but are efficiently catabolised by the gut microorganisms to easily absorbed lower molecular weight compounds [57]. These can reach significant concentrations in tissues: after consumption of almond skins, the peak concentration of phenylacetic acid in human plasma was 8 µM and the 4-OH-3-methoxyphenyl acetic acid in urine 25 µM. The maximum concentrations of most of the microbial catabolites persisted in tissues for 24–43 h, much longer than is common for dietary polyphenols [56,61]. Many of them have aromatic centres and some retain the phenolic groups of their precursors. Not surprisingly, the non-extractable polyphenols have antioxidant, anti-inflammatory and antiatherogenic properties, contributing over 60% to the average antioxidant potential of cereals, legumes and cocoa products [62,63,64]. This, together with the persistence of these metabolites in body fluids, suggests that they provide a steady level of antioxidant protection for hours after a polyphenol-rich meal.

From these observations, the three general conclusions of particular significance for this review are (1) food polyphenols and many of their metabolic derivatives are absorbed by living organisms; (2) combined concentrations of the polyphenols and metabolites in biofluids can reach micromolar levels; and (3) many metabolites of food polyphenols retain their aromatic function sufficient for antioxidant activity.

## 5. Polyphenols in Scavenging of Free Radicals In Vivo

The finding of high rate constants of reactions of aromatic molecules with C-centred free radicals allows an approximate estimate of the potential effectiveness of polyphenols and their aromatic metabolites in delaying, reducing or preventing biological damage under oxidative stress. Since, as already indicated, not all free radicals can or should be scavenged in vivo, the function of added antioxidants is to augment the antioxidant capacity of the organism to a level sufficient to overcome any damage caused by oxidative stress. As the damage initiating primary radicals R^●^ cannot be intercepted, antioxidant repair needs to be applied to the secondary [Target]^●^. Assuming that the two main reactions competing for the [Target]^●^ are as shown in Scheme 2, and using the rate constants of 2 × 10^9^ M^−1^ s^−1^ for the [Target]^●^ reacting with O_2_ and 10^10^ M^−1^ s^−1^ for adduct formation, for 50% of the [Target]^●^ to be repaired, the concentration of the aromatic antioxidants should be 4 µM. Such levels are easily achievable by diets containing the recommended daily amounts of fruits and vegetables and may well be sufficient to neutralise excessive levels of free radicals in oxidative stress. In contrast, a similar efficiency of reactions involving electron or H transfer would require an unachievable antioxidant concentration of ~2 mM and would include only the polyphenols and those of their metabolites retaining the phenolic groups.

## 6. Conclusions and Prospects

The new insight into the mechanism of the free radical–polyphenol reaction provides support for the age-old advice urging consumption of plant-derived foods. Such advice is seldom based on rigorous scientific evidence. The results reviewed here provide evidence that at least part of the demonstrated health benefits of fruit and vegetable diet is likely to be derived from the capacity of the constituent polyphenols to function as antioxidants, reducing or eliminating the ability of free radicals to cause cell and tissue damage. They also identify the basis of the antioxidant properties of a large proportion of polyphenol metabolites and show that they, and other aromatic molecules, are part of the overall antioxidant arsenal of an organism. Besides the plant-derived food components, this aromatic metabolome would include other tissue constituents and many pharmaceuticals used in the treatment of pain or inflammation. New antioxidant drugs with the desirable properties of easy administration, low toxicity and persistence can be developed for individuals, especially those exposed to oxidative stress because of the particular conditions of their lives. Or, one can rely on a varied diet rich in vegetables, fruits, nuts, dark chocolate and red wine.

## Figures and Tables

**Table 1:** Rate constants of repair of the carbon radicals by flavonoids and a metabolite.

C-Radical	Polyphenol		Conditions	10^−7^ × k_f_ (M^−1^s^−1^)	Electron Transfer
Promethazine	EGC		pH 3	1.4	yes
Tryptophan	Catechin		pH 7	2.0	yes
Tryptophan	Quercetin		flavonoid HSA bound	6.8	yes
Tryptophan	Rutin		flavonoid HSA bound	14	yes
Tryptophan	Rutin		micelles	4.8	yes
Tryptophan	Quercetin		micelles	6.0	yes
Uric acid	Quercetin		flavonoid HSA bound	0.2	yes
TMPD	Rutin		pH 13.5	1300	no
Ac-Ala-NH2	Morin		pH neutral	1000	no
Ac-Lys-NH2	Morin		pH neutral	100	no
Ac-Pro-NH2	Morin		pH neutral	>1000	no
Ac-Gly-NH2	Morin		pH neutral	1000	no
Cyclo(Gly)2	Morin		pH neutral	300	no
Ac-Glu-NH2	Morin		pH neutral	>400	no
tert-BuOH	Morin		pH neutral	2000	no
Ac-Ala-NH2	Gallate Rutin EGCG	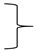	pH neutral	100–1000	no

Values of the rate constants of the forward reactions, k_f_, were derived by pulse radiolysis. ECG, epigallocatechin; EGCG, epigallocatechin gallate; HSA, human serum albumin; TMPD, tetramethyl- *p*-phenylene diamine. Results from References [46,47,48].

## Data Availability

Not applicable.
